# Comparative-effectiveness of vancomycin and linezolid as part of guideline-recommended empiric therapy for healthcare-associated pneumonia

**DOI:** 10.1186/s13104-015-1396-1

**Published:** 2015-09-17

**Authors:** Kelly R. Reveles, Eric M. Mortensen, Russell T. Attridge, Christopher R. Frei

**Affiliations:** College of Pharmacy, The University of Texas at Austin, San Antonio, TX USA; Pharmacotherapy Education and Research Center (PERC), School of Medicine, University of Texas Health Science Center at San Antonio, 7703 Floyd Curl Dr., MSC-6220, San Antonio, TX 78229-3900 USA; The VA North Texas Health Care System, Dallas, TX USA; School of Medicine, The University of Texas Southwestern Medical Center, 5323 Harry Hines, Boulevard, Dallas, TX USA; Feik School of Pharmacy, University of the Incarnate Word, 4301 Broadway, CPO #99, San Antonio, TX USA

**Keywords:** Antibiotic therapy, Guidelines, Pneumonia, Healthcare-associated infections

## Abstract

**Background:**

Linezolid has been directly compared to vancomycin in pneumonia; however, most clinical trials have not compared outcomes specifically in the healthcare-associated pneumonia (HCAP) population. The objective of this study was to compare the effectiveness of vancomycin and linezolid in a national cohort of hospitalized veterans with HCAP.

**Methods:**

This was a retrospective cohort study of Veterans Health Administration patients admitted to >150 hospitals across the United States between 2002 and 2007. Patients were included if they were at least 65 years old, had an ICD-9-CM code for pneumonia, had one or more HCAP risk factors, and received guideline-concordant antibiotic therapy with linezolid or vancomycin within 48 h of admission. Critically ill patients were excluded. Multivariable logistic regression models and propensity scores were used to examine the association between linezolid or vancomycin therapy and 30-day mortality.

**Results:**

A total of 1211 patients met study criteria; 946 received vancomycin and 265 received linezolid. Thirty-day mortality was higher in patients treated with vancomycin (n = 243; 25.7 %) as compared to linezolid (n = 33; 12.5 %) (adjusted OR 2.56; 95 % CI 1.67–4.04). Vancomycin use (n = 945) was also predictive of 30-day mortality compared to linezolid use (n = 264) in the propensity score analysis (adjusted OR 2.55; 95 % CI 1.66–4.02).

**Conclusion:**

Linezolid was associated with decreased patient mortality compared to vancomycin in a national cohort of non-critically ill, hospitalized veterans with HCAP.

## Background

In the United States, pneumonia is the second leading cause of hospitalization and is responsible for 5.6 million infections and 1.2 million hospitalizations annually [1, 2]. Healthcare-associated pneumonia (HCAP) is an important subgroup of pneumonia in which community-dwelling patients have had recent exposure to the health care system. These patients are more likely to be infected with multi-drug resistant pathogens, such as methicillin-resistant *Staphylococcus aureus* (MRSA), and suffer poorer health outcomes compared to patients classified as community-acquired pneumonia (CAP) [3]. Appropriate empirical antimicrobial therapy directed against the most likely pathogens has been correlated with improved clinical outcomes in nosocomial pneumonia [4]. The 2005 American Thoracic Society/Infectious Diseases Society of America (ATS/IDSA) guidelines recommend empiric triple-drug therapy for HCAP patients to include double-coverage for *Pseudomonas* and anti-MRSA coverage with vancomycin or linezolid [5].

Vancomycin has been the drug of choice for MRSA pneumonia for many years; however, vancomycin has poor penetration into the lungs and may be associated with renal toxicity [6, 7]. Reports of vancomycin heteroresistance are increasing [8] and there are concerns for clinical failure with vancomycin in the setting of nosocomial pneumonia [8]. Linezolid is an attractive alternative to vancomycin because of its increased lung penetration and minimal risk of renal adverse events [9, 10]. Linezolid has been directly compared to vancomycin in pneumonia; however, most clinical trials have not compared outcomes specifically in the HCAP population. Therefore, the primary objective of this study was to compare 30-day mortality in a national cohort of non-critically ill veterans treated with guideline-concordant HCAP therapy with either linezolid or vancomycin. Secondary objectives included a comparison of 60 and 90-day mortality.

## Methods

### Study design

This was a population-based cohort study in adult veterans receiving care from the Veterans Health Administration (VHA) between fiscal years 2002 and 2007. This study was approved by the Institutional Review Board of The University of Texas Health Science Center at San Antonio and the South Texas Veterans Health Care System Research and Development committee. A description of the methods used to build this data resource has been previously reported [11].

### Data source

The VHA is the largest integrated health care system in the United States and includes more than 150 VHA hospitals and 850 VHA clinics. Data for this study were obtained from the VHA electronic medical record system which includes administrative, clinical, laboratory, and pharmacy data repositories. Four national VA data sources were utilized: the VA Medical SAS Datasets (both inpatient and outpatient), the VA Vital Status File, the VA Decision Support System Datasets, and the VHA Annual Enrollment Files.

### Patient eligibility

**Fig. 1:**
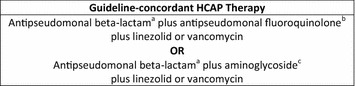
Definition of guideline-concordant HCAP therapy [5], *HCAP* healthcare-associated pneumonia, ^a^Antipseudomonal β-lactams include cefepime, ceftazidime, imipenem-cilastatin, meropenem, piperacillin-tazobactam and ticarcillin-clavulanate (aztreonam may be substituted in penicillin-allergic patients), ^b^Antipseudomonal fluorquinolones include levofloxacin and ciprofloxacin, ^c^Aminoglycosides include gentamicin, tobramycin, or amikacin

Patients were included in this study if they: (1) were at least 65 years old, (2) were hospitalized between fiscal years 2002 and 2007, (3) had an *International Classification of Diseases, 9th Revision, Clinical Modification* (ICD-9-CM) principal discharge diagnosis of pneumonia (480.0-483.99 or 485-487) or a secondary discharge diagnosis with a principal diagnosis of respiratory failure (ICD-9-CM code 518.81) or sepsis (ICD-9-CM code 038.xx), and 4) had at least one documented HCAP risk factor as defined by the ATS/IDSA guidelines [5] (hospital admission in the previous 90 days, residence in a nursing home in the previous 90 days, receipt of outpatient intravenous antibiotics in the previous 90 days, or hemodialysis). Patients were also required to have received triple-drug antibiotic therapy, in accordance with HCAP guidelines, within the first 48 h of hospital admission (Fig. 1). This definition was included to minimize the number of patients with nosocomial pneumonia included in the study. Only unique patients, with a single hospital admission, over the study period were included. Critically ill patients were excluded from the analysis to minimize differences in HCAP severity between treatment groups. Critical illness was defined by: (1) admission to the intensive care unit (ICU), (2) ICD-9-CM codes for respiratory organ failure, cardiovascular organ failure, or invasive mechanical ventilation, or (3) vasopressor therapy or inotrope therapy (dopamine, dobutamine, epinephrine, isoproterenol, metaraminol, norepinephrine, and vasopressin).

### Baseline characteristics

Patient demographics, comorbid conditions, HCAP risk factors, and medication use were characterized at the time of hospital admission. Year of hospitalization was dichotomized into years 2001 through 2004 and 2005 through 2007 to facilitate statistical analyses. Race was recorded as Black or White; Native American, Hawaiians, and those with missing race were grouped into an “other” category. Patients identifying themselves as “Hispanic” were also categorized. VHA priority group was characterized for all patients. Priority group has been associated with severity of illness and socioeconomic status, and has been validated with VHA administrative data [12, 13]. Comorbid conditions were classified using ICD-9-CM codes and are presented individually as well as in composite using the age-adjusted Charlson Comorbidity Score [14]. HCAP risk factors and medication use within 90 days of admission were identified using electronic inpatient and outpatient medical records. Tobacco use was defined as a diagnosis of nicotine dependence (current procedural terminology code 99406 or 99407) or a prescription for a smoking cessation product (e.g., Zyban^®^, varenicline, Nicotrol^®^, or nicotine replacement). Patients with alcohol abuse or invasive mechanical ventilation were identified using ICD-9-CM codes. Finally, organ failure included any neurological, renal, hematological, or hepatic organ failure, as defined by ICD-9-CM codes.

### Antibiotic therapy and pathogens

Antibiotic therapy was characterized within the first 48 h of hospital admission. Those patients who received guideline-concordant HCAP therapy, as defined by the consensus guidelines [5], were further divided into groups depending on the anti-MRSA therapy they received (vancomycin or linezolid). Patients who received both linezolid and vancomycin were excluded from analysis. There was no minimum number of doses of vancomycin or linezolid for study inclusion. Pathogens were identified using ICD-9-CM codes. The following pathogens were queried: *Staphylococcus aureus*, methicillin-resistant *S. aureus* (MRSA), *Streptococcus pneumoniae*, *Streptococcus* other, *Pseudomonas, Klebsiella pneumoniae*, *Escherichia coli*, *Haemophilus influenzae*, other Gram-negative pathogens, *Mycoplasma, Legionella*, *Chlamydia,* and anaerobes.

### Mortality

The primary outcome of this study was 30-day mortality. This outcome measure has been demonstrated to be more closely associated with pneumonia-related mortality compared to 60 or 90-day mortality, which are primarily related to other comorbid conditions [15]. Secondary outcomes included 60-day mortality and 90-day mortality. Mortality was assessed using the VHA Vital Status File, which has a similar sensitivity and specificity as compared to the “gold standard” National Death Index [16].

### Data and statistical analysis

All statistical analyses were conducted using JMP 9.0^®^ (SAS Corp, Cary, NC, USA). Prior to multivariable analyses, all study measures were evaluated using appropriate two-way statistical tests to describe the sample. Categorical variables were presented as the number and percentage of subjects in each category and were compared using the Chi square test. Continuous variables were presented as medians and interquartile ranges (IQRs) and were compared using the Wilcoxon rank sum test, as all variables were non-normally distributed.

We utilized multivariable logistic regression models to evaluate the association of linezolid and vancomycin use and 30, 60, and 90-day mortality. Multivariable logistic regression aims to determine the effect of an exposure (e.g., linezolid versus vancomycin), while holding other covariates constant. Baseline characteristics that resulted in a *p* value <0.10 in bivariable analyses between linezolid and vancomycin groups were entered simultaneously into the final model as covariates. These explanatory variables included: year of hospitalization (binary variable; 2001–2004 and 2005–2007), race, Hispanic ethnicity, VHA priority group, tobacco use, hospital admission in the previous 90 days, outpatient intravenous antibiotics in the previous 90 days, myocardial infarction, congestive heart failure, chronic obstructive pulmonary disease, tobacco use, cardiovascular medications, anti-diabetic medications, inhaled corticosteroids, systemic corticosteroids, pulmonary medications, organ failure, MRSA, and *Streptococcus pneumoniae*. We found that the presence of more than one HCAP risk factor was predictive of mortality in our previous study and, thus, included it as a covariate in this model.

To further reduce bias in our non-randomized cohort, we conducted a second set of analyses whereby we calculated a propensity score for the receipt of linezolid versus vancomycin. The propensity scores were determined using a logistic regression model with treatment group as the dependent variable and all of the explanatory variables listed above as covariates. Patients whose propensity scores did not fall within the overlapping common support region were excluded from propensity score analyses. We calculated the area under the receiver-operating-characteristic curve to assess the discriminatory power of the propensity score model. Then, we constructed multivariable logistic regression models to evaluate the association of linezolid and vancomycin use and 30, 60, and 90-day mortality. In each of these models, we included the propensity score and all other explanatory variables as covariates.

## Results

### Overall population

Overall, we identified 62,682 patients with pneumonia. After applying exclusion criteria, 1211 patients had at least one HCAP risk factor and had received guideline-concordant HCAP therapy with vancomycin (n = 946) or linezolid (n = 265) within 48 h of hospital admission.

Table 1 describes the patients’ baseline characteristics. Patients were predominately elderly (median age of 76 years), male (98 %), and White (79 %). The most common HCAP risk factor was hospitalization within 90 days (78 %). Baseline characteristics differed between linezolid and vancomycin-treated patients with respect to year of hospitalization, race, ethnicity, VHA priority group, and certain comorbid conditions (Table 1).

**Table 1:** Baseline characteristics in HCAP patients treated with vancomycin or linezolid

	Overall	Linezolid	Vancomycin	P-value^a^
Patients, n	1211	265	946	N/A
Year of hospitalization, n (%)				
2001–2004	541 (44.7)	153 (57.7)	388 (41.0)	<*0.0001*
2005–2007	670 (55.3)	112 (42.3)	558 (59.0)	
Age (years), median (IQR)	76 (70–80)	76 (70–80)	76 (70–81)	0.892
Male, n (%)	1189 (98.2)	259 (97.7)	930 (98.3)	0.537
Race, n (%)				
White	961 (79.4)	225 (84.9)	736 (77.8)	*0.033*
Black	188 (15.5)	32 (12.1)	156 (16.5)	
Other	62 (5.1)	8 (3.0)	54 (5.7)	
Hispanic ethnicity, n (%)	159 (13.1)	10 (3.8)	149 (15.7)	<*0.0001*
VHA priority group, n (%)				
1	280 (23.1)	45 (17.0)	235 (24.8)	*0.019*
2–6	859 (70.9)	200 (75.5)	659 (69.7)	
7–8	72 (6.0)	20 (7.5)	52 (5.5)	
HCAP risk factors, n (%)				
Hospitalization within 90 days	941 (77.7)	191 (72.1)	750 (79.3)	*0.013*
Nursing home resident within 90 days	36 (3.0)	7 (2.6)	29 (3.1)	0.719
Hemodialysis	514 (42.4)	112 (42.3)	402 (42.5)	0.947
Outpatient IV antibiotics within 90 days	152 (12.6)	22 (8.3)	130 (13.7)	*0.018*
>1 HCAP risk factor, n (%)	384 (31.1)	62 (23.4)	322 (34.0)	*0.001*
Charlson comorbidity score, median (IQR)	4 (2–6)	4 (2.5–5.5)	4 (2-6)	0.233
Comorbid conditions, n (%)				
Myocardial infarction	121 (10.0)	34 (12.8)	87 (9.2)	0.081
Heart failure	381 (31.5)	103 (38.8)	278 (29.4)	*0.003*
Cerebrovascular disease	302 (24.9)	59 (22.3)	243 (25.7)	0.255
Chronic obstructive pulmonary disease	614 (50.7)	164 (61.9)	450 (47.6)	<*0.0001*
Liver disease	16 (1.3)	1 (0.4)	15 (1.6)	0.128
Chronic kidney disease	523 (43.2)	114 (43.0)	409 (43.2)	0.950
Diabetes	442 (41.6)	90 (39.0)	352 (42.2)	0.376
Neoplastic disease	433 (35.8)	92 (34.7)	341 (36.0)	0.690
HIV/AIDS	4 (0.3)	1 (0.4)	3 (0.3)	0.880
Substance abuse or dependence, n (%)				
Tobacco use	401 (33.1)	100 (37.7)	301 (31.8)	0.070
Alcohol abuse or dependence	49 (4.0)	11 (4.2)	38 (4.0)	0.922
Medication use within 90 days, n (%)				
Cardiovascular medications	796 (65.7)	196 (74.0)	600 (63.4)	*0.001*
Anti-diabetic medications	309 (25.5)	78 (29.4)	231 (24.4)	0.098
Inhaled corticosteroids	236 (19.5)	67 (25.3)	169 (17.9)	*0.007*
Systemic corticosteroids	321 (26.5)	87 (32.8)	234 (24.7)	*0.008*
Pulmonary medications	431 (35.6)	123 (46.4)	308 (32.6)	<*0.0001*
Noninvasive mechanical ventilation, n (%)	24 (2.0)	3 (1.1)	21 (2.2)	0.262
Organ failure, n (%)	266 (22.0)	45 (17.0)	221 (23.4)	*0.027*
Pathogens with a prevalence ≥1 %, n (%)				
*Staphylococcus aureus*	87 (7.2)	4 (1.5)	83 (8.8)	*0.019*
Methicillin-resistant *S. aureus*	66 (5.5)	5 (1.9)	61 (5.0)	*0.004*
*Streptococcus pneumoniae*	32 (2.6)	8 (3.0)	24 (2.5)	*0.005*
*Pseudomonas*	55 (4.5)	7 (2.6)	48 (5.1)	0.569
*Klebsiella pneumoniae*	16 (1.3)	0 (0.0)	16 (1.7)	0.152

*HCAP* healthcare-associated pneumonia

^a^Italics indicates statistical significance

Overall, only 225 patients (18.6 %) received an ICD-9-CM code for a bacterial pathogen, predominately with a single organism (15.9 %). *S. aureus* was the most commonly isolated pathogen (7.2 %), of which 5.5 % were MRSA. MRSA was identified in a significantly higher proportion of vancomycin-treated patients (7.3 versus 2.2 %, p = 0.02).

### Mortality

**Fig. 2:**
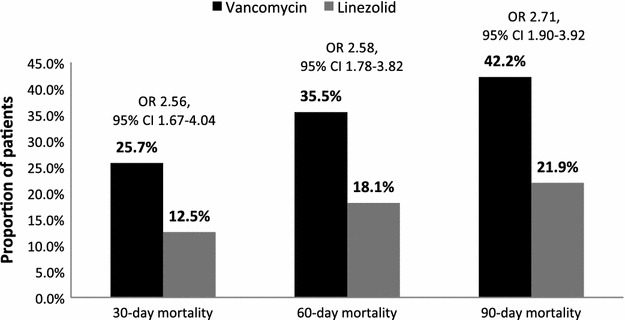
Median 30, 60, and 90-day mortality in HCAP patients treated with vancomycin or linezolid, *HCAP* healthcare-associated pneumonia, *OR* odds ratio, *CI* confidence interval. The figure depicts crude mortality rates. The adjusted odds ratios and 95 % confidence intervals were derived from multivariable logistic regression models; see text for covariates

Overall, 30-day mortality, 60-day mortality, and 90-day mortality were 22.8, 31.5, and 37.8 %, respectively. Crude 30-day mortality was higher in patients treated with vancomycin (25.7 %) compared to linezolid (12.5 %) (Fig. 2). After adjustment for potential confounders, vancomycin use was a significant predictor of 30-day mortality (adjusted OR 2.56; 95 % CI 1.67–4.04). Similarly, vancomycin use was associated with higher 60-day mortality (adjusted OR 2.58; 95 % CI 1.78–3.82) and 90-day mortality (adjusted OR 2.71; 95 % CI 1.90–3.92) compared to linezolid. Prior hospital admission and year of hospitalization were also predictive of 30-day mortality (Table 2).

**Table 2:** Risk factors for 30-day mortality in patients treated with vancomycin or linezolid

Risk factors	Adjusted OR (95 % CI)^a,b^
Vancomycin versus linezolid use	*2.56 (1.67*–*4.04)*
Year of hospitalization, 2005-2007	*0.61 (0.44*–*0.83)*
Race (White)	1.13 (0.66–1.90)
Race (Black)	1.23 (0.66–2.49)
Hispanic ethnicity	0.64 (0.39–1.01)
VHA priority group 1	1.35 (0.71–2.52)
VHA priority group 2–6	0.67 (0.34–1.34)
Prior hospital admission	*2.58 (1.66*–*4.12)*
Outpatient intravenous antibiotics	1.00 (0.60–1.64)
>1 HCAP risk factor	0.78 (0.53–1.15)
Myocardial infarction	1.02 (0.60–1.67)
Congestive heart failure	0.78 (0.55–1.12)
Chronic obstructive pulmonary disease	0.84 (0.59–1.21)
Smoking	0.84 (0.60–1.18)
Cardiovascular medications	1.04 (0.74–1.47)
Anti-diabetic medications	1.07 (0.74–1.54)
Inhaled corticosteroids	0.63 (0.38–1.01)
Systemic corticosteroids	0.73 (0.49–1.07)
Pulmonary medications	1.40 (0.94–2.10)
Organ failure	1.18 (0.80–1.73)
Methicillin-resistant *S. aureus*	0.68 (0.34–1.27)
*Streptococcus pneumoniae*	0.75 (0.27–1.80)

*HCAP* healthcare-associated pneumonia, *VHA* Veterans Health Administration, *OR* odds ratio, *CI* confidence interval

^a^Results of multivariable logistic regression model; see text for covariates

^b^Italics indicates statistical significance

The area under the receiver-operating characteristic curve for the propensity score derivation model was 0.67. After excluding patients with non-overlapping propensity scores, 945 vancomycin-treated patients and 264 linezolid-treated patients remained for propensity score analyses. When propensity scores were included in the logistic regression model, vancomycin use remained a significant predictor of 30-day mortality (adjusted OR 2.55; 95 % CI 1.66–4.02), 60-day mortality (adjusted OR 2.60; 95 % CI 1.79–3.84), and 90-day mortality (adjusted OR 2.70; 95 % CI 1.89–3.91). Our post hoc statistical analysis calculated to 99.9 % power for comparisons between vancomycin and linezolid treatment groups.

## Discussion

Our study suggests that patients with HCAP may benefit from guideline-concordant triple-drug therapy that contains linezolid as compared to vancomycin. To our knowledge, this is the first study to demonstrate improved clinical outcomes associated with linezolid use in an HCAP population.

Other studies have evaluated the effectiveness of linezolid and vancomycin in nosocomial pneumonia; however, comparisons in HCAP are limited. In a recently published trial, Wunderink and colleagues [10] compared the clinical effectiveness of linezolid to vancomycin in MRSA nosocomial pneumonia. This study demonstrated improved clinical cure with linezolid compared to vancomycin; however, there was no difference in 60-day mortality between treatment groups. In a subgroup analysis of HCAP patients, linezolid-treated patients had higher cure rates than vancomycin-treated patients; however, the comparison failed to achieve statistical significance, possibly due to the limited sample size in the HCAP subgroup (n = 54). Additionally, mortality was not reported in the HCAP subgroup. The 60-day mortality rate in our cohort overall was 31.5 %. Our 60-day mortality in the linezolid group (18.1 %) was similar to that seen by Wunderink and colleagues (15.7 %); however, vancomycin-treated patients in our study suffered higher 60-day mortality (35.3 versus 17.0 %) as compared to that study. Increased median age in our population (76 versus 61 years) may partially explain the higher 60-day mortality, as well as the VA population which often has more comorbidities and poorer health than the community hospital population.

Our study adds to the findings of Wunderink and colleagues by examining a much larger HCAP population to provide sufficient sample size to study important HCAP clinical outcomes. Although our study was observational, the use of multivariable models enables us to account for potential confounding variables that may affect patient outcomes. Additionally, compared to randomized controlled trials, our study population might more closely resemble the general population. Furthermore, our study investigated 30-day mortality, which has been more closely related to pneumonia-related mortality as compared to the 60-day mortality endpoint used in clinical trials. These factors could have led to different outcomes in our study as compared to trials.

In addition to Wunderink et al., other studies have compared clinical outcomes between linezolid and vancomycin-treated patients in nosocomial pneumonia; however, results have been inconsistent. While some studies have demonstrated improved clinical outcomes favoring linezolid treatment [10, 17], others did not show a significantly improved clinical benefit with linezolid compared to vancomycin [18, 19]. Two recent meta-analyses found similar clinical outcomes with linezolid compared to vancomycin in MRSA nosocomial pneumonia [20]. Our study indicates that HCAP patients are an important subgroup of pneumonia patients who may benefit from initial treatment with linezolid as compared to vancomycin.

There were several baseline characteristics that differed between linezolid and vancomycin-treated patients that are worth noting. First, vancomycin was used more frequently in the latter half of the study period. This could be due to linezolid restrictions within the VA or increased understanding of dosing and administration of vancomycin in more recent years. The years 2005–2007 were found to be predictive of lower mortality compared to prior years. This might reflect improvements in the care for patients with pneumonia overall in more recent years. Next, recent hospital admission was associated with a 2.5-fold increase in mortality, while controlling for a number of severity indicators and chronic comorbid conditions. While we cannot determine the specific cause in this study, we hypothesize that this might be related to poorer overall health status that would necessitate frequent hospital admissions. Lastly, although not significantly different in multivariable models, bivariable models indicated that linezolid was used more often in White, non-Hispanic patients. This might be related to socioeconomic factors, but we were unable to determine this association in our study.

Most of the studies evaluating outcomes between linezolid and vancomycin in nosocomial pneumonia have used a patient population consisting only of culture-positive patients. In our study, only 18.6 % of patients were culture positive. There were too few patients with identified bacterial pathogens to compare outcomes between linezolid and vancomycin; however, the use of propensity scores allowed us to control for pathogen differences between the linezolid and vancomycin groups. Because an organism is often not identified in HCAP, it is important to have appropriate empiric therapy targeted to the most likely pathogens [5]. Numerous studies have demonstrated that early appropriate antibiotic therapy is associated with improved outcomes in pneumonia [4, 21, 22]. Our study provides evidence that using linezolid as part of the empiric regimen may lead to more favorable patient outcomes.

There are several factors that may influence the effectiveness or favorability of linezolid as compared to vancomycin in pneumonia. These include: (1) pharmacokinetics, (2) altered pharmacodynamics, and (3) toxicity. Pharmacokinetic studies have demonstrated that vancomycin lung epithelium concentrations are approximately two-fifths of that in the plasma [23, 24]. This could result in inadequate lung concentrations to achieve levels above the organism’s minimum inhibitory concentration (MIC). Second, studies have correlated increasing MICs to clinical failure in MRSA bacteremia and pneumonia treated with vancomycin [8, 25]. Linezolid inhibits protein synthesis at an early stage of bacterial replication, thus cross-resistance to other antimicrobials is thought to be limited [26]. Finally, vancomycin has been associated with adverse effects on the kidneys, especially when combined with other nephrotoxic agents recommended for HCAP treatment (e.g., aminoglycosides) [7, 27].

This study has potential limitations. The study utilized a national cohort of veterans consisting of a primarily elderly male population; therefore, these results may not be generalizable to non-VHA settings. Veterans who sought care at non-VHA hospitals would not be captured in our cohort. We used ICD-9-CM codes to define several variables. Use of ICD-9-CM coding is often necessary for data collection in large databases to identify a sufficient sample size; however, ICD-9-CM coding may contain errors. Despite this, there are data demonstrating favorable positive and negative predictive values (86 and 97 %, respectively) in pneumonia [28]. The VA Vital Status File has high sensitivity (~98 %) for capturing mortality [16], but it is possible that mortality may have been missed in some patients. The cause of death was not available, so we were unable to attribute death specifically to pneumonia. Other factors may have influenced outcomes in our population. We attempted to limit differences in severity of illness between groups by excluding critically ill patients and by controlling for other factors, such as comorbidities and medication use, in our multivariable models. However, other factors (e.g., CURB-65 score, pneumonia severity index, baseline serum creatinine, individual organ failures, pH, blood urea nitrogen, hematocrit, hospital readmission) may have varied among patients within each group and influenced mortality rates. It is also possible that other unmeasured patient baseline characteristics differed between groups in the propensity score model, but we were unable to assess this since the groups were not matched by propensity score. Treatment-related factors, such as prior vancomycin use, antimicrobial failure, switch therapy, targeted therapy following pathogen detection, vancomycin blood levels, drug toxicities, and length of therapy could not be accounted for in our study design. Specifically, optimized vancomycin dosing to achieve adequate serum and tissue concentrations is important to microbiogical cure and other clinical outcomes. Under-dosing of vancomycin, though not evaluated in this study, could have affected mortality rates. These treatment-related factors were not evaluated due to lack of information in the medical chart or the need for a manual chart review, which was precluded by our sample size. Furthermore, pathogens were identified by ICD-9-CM code; therefore, we could not confirm diagnosis from microbiological analysis or determine pathogen minimum inhibitory concentrations. Given the low rate of culture-positivity in this population, the proportion of MRSA identified through culture might not reflect the overall prevalence of MRSA. Physician preferences and other unmeasured factors may have influenced the decision to initiate one antimicrobial agent over another; however, we were unable to determine these associations with our study design. Clinicians might not use guideline-concordant triple antibiotic therapy for HCAP, though this definition was used to limit differences in severity of illness and antimicrobial coverage between groups. Finally, we limited our cohort to non-critically ill patients with a diagnosis of pneumonia; therefore, our results may not be generalizable to patients with severe HCAP.

We acknowledge that the current guidelines recommending triple-drug therapy for HCAP patients have yet to be validated [11, 29]; however, we used guideline-concordant therapy as part of our inclusion criteria to ensure adequate antimicrobial coverage for pathogens that have been associated with HCAP and to limit the differences in antimicrobial coverage to the anti-MRSA agent. Furthermore, use of the HCAP criteria might not adequately predict resistant pathogens [30]. Further study of the optimal treatment regimen for HCAP patients is necessary to improve outcomes in this unique population.

## Conclusion

Linezolid use was associated with decreased patient mortality compared to vancomycin use in a national cohort of elderly, non-critically ill, hospitalized veterans with HCAP.
